# Life style and interaction with microbiota in prostate cancer patients undergoing radiotherapy: study protocol for a randomized controlled trial

**DOI:** 10.1186/s12885-022-09521-4

**Published:** 2022-07-19

**Authors:** Patrizia Gnagnarella, Giulia Marvaso, Barbara Alicja Jereczek-Fossa, Ottavio de Cobelli, Maria Claudia Simoncini, Luiz Felipe Nevola Teixeira, Annarita Sabbatini, Gabriella Pravettoni, Harriet Johansson, Luigi Nezi, Paolo Muto, Valentina Borzillo, Egidio Celentano, Anna Crispo, Monica Pinto, Ernesta Cavalcanti, Sara Gandini, for the MicroStyle Collaborative Group, Costanza Gavioli, Silvia Ciceri, Marialetizia Latella, Giulia Corrao, Dario Zerini, Debora Macis, Valentina Aristarco, Gabriele Cozzi, Ketti Mazzocco, Fodor Cristiana Iuliana, Serena Galiè, Carlotta Catozzi, Rossella Di Franco, Nunzio De Martino, Maria Grimaldi, Concetta Montagnese, Melania Prete, Flavia Nocerino, Emanuela Rotondo, Sergio Arpino, Serena Meola, Francesco Labonia, Federica Bellerba

**Affiliations:** 1 Division of Epidemiology and Biostatistics, European Institute of Oncology IRCSS, Milan, Italy; 2 Department of Radiation Oncology, European Institute of Oncology IRCSS, Milan, Italy; 3 Department of Oncology and Hemato-oncology, University of Milan, Milan, Italy; 4 Division of Urology, European Institute of Oncology IRCSS, Milan, Italy; 5 Physiotherapy Unit, European Institute of Oncology IRCSS, Milan, Italy; 6 Dietetic and Clinical Nutrition Unit, European Institute of Oncology IRCSS, Milan, Italy; 7 Applied Research Division for Cognitive and Psychological Sciences, European Institute of Oncology IRCSS, Milan, Italy; 8 Division of Cancer Prevention and Genetics, European Institute of Oncology IRCSS, Milan, Italy; 9 Department of Experimental Oncology, European Institute of Oncology IRCSS, Milan, Italy; 10 Department of Radiation Oncology, Istituto Nazionale Tumori-IRCCS-Fondazione G. Pascale, Naples, Italy; 11 Epidemiology and Biostatistics Unit, Istituto Nazionale Tumori-IRCCS-Fondazione G. Pascale, Naples, Italy; 12 Rehabilitation Medicine Unit, Strategic Health Services Department, Istituto Nazionale Tumori-IRCCS-Fondazione G. Pascale, Naples, Italy; 13 Laboratory Medicine Unit, Istituto Nazionale Tumori-IRCCS-Fondazione G. Pascale, Naples, Italy

**Keywords:** Prostate cancer, Randomized controlled trial, Diet, Physical activity, Counseling, Quality of life, Body composition, Microbiome, Serum biomarkers, Radiotherapy

## Abstract

**Background:**

Prostate cancer (PCa) is the second most common cancer in men worldwide. The standard non-surgical approach for localized PCa is radiotherapy (RT), but one of the limitations of high-dose RT is the potential increase in gastrointestinal and genitourinary toxicities. We present the protocol of the Microstyle study, a multicentre randomized two-arm crossover clinical trial. The primary outcome will be assessed at the end of 6-month intervention, by measuring the change in adherence to a healthy lifestyle score. The hypothesis is that modifying lifestyle we change microbiome and improve quality of life and decrease side effects of RT.

**Methods:**

Study participants will be recruited among men undergoing RT in two Italian centers (Milan and Naples). We foresee to randomize 300 patients in two intervention arms: Intervention Group (IG) and Control Group (CG). Participants allocated to the IG will meet a dietitian and a physiotherapist before RT to receive personalized diet and exercise recommendations, according to their health status, to improve overall lifestyle and reduce side effects (bowel and/or urinary problems). Dietitian and physiotherapist will work together to set individualized goals to reduce or eliminate side effects and pain according to their health status. All participants (IG) will be given a pedometer device (steps counter) in order to monitor and to spur participants to increase physical activity and reduce sedentary behavior. Participants included in the CG will receive baseline general advice and materials available for patients undergoing RT. According to the cross-over design, the CG will cross to the intervention approach after 6-month, to actively enhance compliance towards suggested lifestyle recommendations for all patients.

**Discussion:**

This trial is innovative in its design because we propose a lifestyle intervention during RT, that includes both dietary and physical activity counselling, as well as monitoring changes in microbiome and serum biomarkers. The promotion of healthy behaviour will be initiated before initiation of standard care, to achieve long lasting effects, controlling side effects, coping with feelings of anxiety and depression and improve efficacy of RT.

**Trial registration:**

ClincalTrial.gov registration number: NCT05155618. Retrospectively registered on December 13, 2021. The first patient was enrolled on October 22, 2021.

**Supplementary Information:**

The online version contains supplementary material available at 10.1186/s12885-022-09521-4.

## Background

Prostate cancer (PCa) is the second most frequent cancer and the fifth leading cause of cancer death among men in 2020, worldwide [1]*.* The standard non-surgical approach for localized PCa is radiotherapy (RT) which might causes acute and late gastrointestinal and genitourinary toxicity [2]. The technological improvements of the last decades and the use of Intensity-Modulated RT (IMRT) allowed reducing the amount of potentially toxic high doses to rectum and urinary bladder [2, 3].

Acute toxicities, such as diarrhea, dysuria and nausea, could develop after 2 to 3 weeks of RT and continue to occur for several weeks or months following treatment completion [4, 5]. PCa patients may experience weight loss attributable to radiation side effects, which can affect appetite and in the long term the nutritional status [6, 7]. Changes in body weight and composition can compromise treatment accuracy and increase toxicity because it affects RT dose distribution increasing dose received by healthy tissues [6]. Furthermore, increasing age, time since diagnosis and comorbidities amplify physical morbidity, poor symptom control, high perceived fatigue and in general a poor health-related quality of life (QoL), as well as psychosocial concerns (e.g., mood changes, distress) [8, 9]. Nutritional status is pivotal to manage not only fatigue and quality of life (QoL) [10, 11], but also to reduce PCa-specific mortality [12, 13].

Several studies suggest that nutritional intervention can have a positive effect on toxicities, weight control and QoL in PCa patients [14–16]. No firm conclusion has been drawn on the efficacy of dietary modifications [17, 18], but individualized approach based on appropriate professional counselling to manipulate dietary intake based on emerging symptoms throughout treatment is desirable [19].

At the same time, physical activity has shown to be safe and feasible in cancer patients [20–22], because it seems to be effective to maintain and improve muscle mass, cardiorespiratory fitness, function of the immune system, self-esteem, mood and QoL [23–25]. Moreover, physical activity appear to have a positive effect on cancer related fatigue, the most frequently reported side effect of cancer treatment [26, 27]. Cancer related fatigue is characterized by sleep dysfunction, muscle weakness, mood disturbance and cognitive impairments and it can have a negative influence on QoL in cancer patients.

The number of studies to evaluate the effect of dietary and/or exercise in PCa patients have increased in recent years [17, 28–31]. However, these studies are not designed to evaluate the combined effect of dietary changes combined with exercise in PCa patients undergoing RT, nor to elucidate their effects on gut microbiota and RT-toxicity.

Gut microbiota seems to be associated with gastrointestinal toxicities and have the potential to predict RT-induced toxicities and QoL in patients undergoing this treatment [32–34]. Few studies showed that RT-associated toxicity can be predetermined based on gut microbiota profile in PCa patients [35, 36]. The rate of acute Grade ≥ 2 rectal toxicity is about 20%. The 5-year Grade ≥ 2 risks for rectal bleeding, urgency/tenesmus, diarrhea, and fecal incontinence are 9.9, 4.5, 2.8, and 0.4%, respectively [37]. More recently, Reis Ferreira [38] reported the largest clinical study evaluating the associations between microbiota and acute and late radiation enteropathy in three cohorts of patients undergoing pelvic RT. They conclude that RT may upset the balance of microbiota, by decreasing the influence of key microorganisms, probably more susceptible to radiation effects. They observed a trend for higher pre-RT diversity in patients with no self-reported symptoms and diversity decreased less over time in patients with rising radiation enteropathy. Higher counts of *Clostridium* IV, *Roseburia*, and *Phascolarcto* bacterium were significantly associated with radiation enteropathy. Homeostatic intestinal mucosa cytokines related to microbiota regulation and intestinal wall maintenance were significantly reduced in radiation enteropathy (IL7, IL12/IL23p40, IL15 and IL16) [38].

No comprehensive analyses have been performed to investigate the influence of irradiation on gut microbiota in PCa patients and whether diet and physical activity may have a role in improving QoL modifying microbiome and serum biomarkers. In our previous case–control study, we found that diet, microbiome, vitamin D, markers of inflammation and adipokines are strongly connected in a complex network, and that the unbalance of one or more factors may contribute to colorectal cancer incidence and prognosis [39]. Moreover, we investigated the relation between diet, lifestyle and QoL among breast cancer survivors in a multi-arm clinical trial (InForma), with the support of a motivational approach and the use of a pedometer device to provide important insights regarding the most effective approach in promoting weight loss in overweight and obese breast cancer survivors [40].

Given the above considerations, we present a multicentre randomized two-arm crossover clinical trial to evaluate the impact of 6-month intervention in a group of PCa patients undergoing RT, to address the mechanism(s) by which microbiome may shape effect of the lifestyle intervention on both radiotherapy toxicities and efficacy.

## Methods/design

### Aim, design and setting of the study

**Fig. 1:**
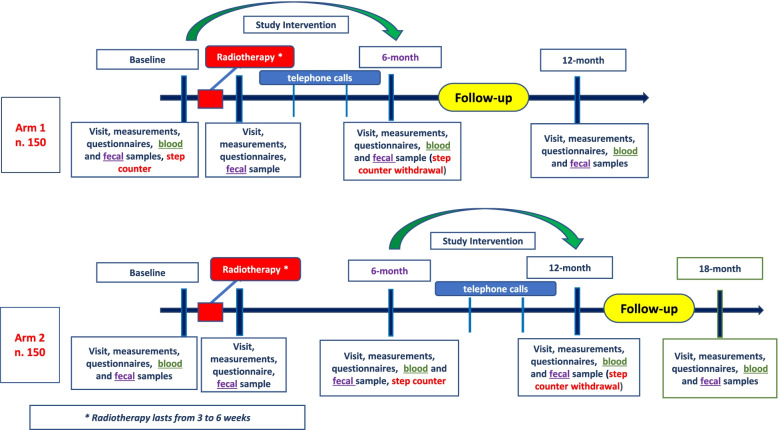
Study design

**Fig. 2:**
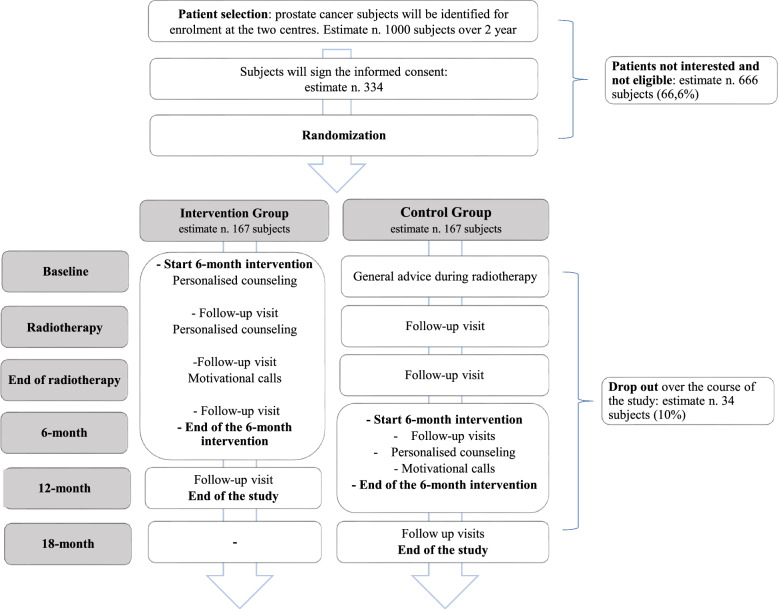
Flow chart

Microstyle (**Micro**biota and life-**Style** in prostate cancer patients undergoing radiotherapy) is a multicentre randomized controlled trial. The present research aims to evaluate the impact of 6-months intervention by measuring the change in adherence to a healthy lifestyle score in a group of PCa patients undergoing RT and to address the mechanism(s) by which intestinal bacteria may shape effect of the dietary intervention on both RT toxicities and efficacy. During a 12–18 months period, randomized patients will receive a 6-months intervention and will be followed for other 6-months (Fig. 1). The crossover design helps in reducing drop-out and to offer all patients the same opportunities, and also to evaluate the effect of the intervention after 6-month from RT when patients should have recreated a healthier microbiome and have less treatment side effects (Fig. 2).

### Study population

#### Participant characteristics

Potential study participants will be recruited among non-metastatic PCa patients undergoing RT. It is envisaged that 334 patients will be enrolled (Fig. 2) to obtain a final sample of 300. Study participants will be recruited and enrolled in two centers, at the Division of Radiation Oncology at European Institute of Oncology (IEO), Milan and Department of Radiation Oncology, at the National Cancer Institute, “Fondazione G. Pascale”, Naples.

#### Eligibility criteria

The study will be open to men aged 18 or older, candidates for prostate treatment with RT (which includes exclusive RT +/−hormone therapy, adjuvant or salvage RT +/− hormone therapy), presenting good performance status (Eastern Cooperative Oncology Group Performance Status Scale - ECOG PS < 2). Only men willing to be randomized to either group and to wear the wrist-based activity monitor during the 6-months study period, will be enrolled. Exclusion criteria will be the following: body mass index (BMI) < 18.5, extra pelvic lymph node involvement or metastasis, malnutrition according to the Malnutrition Universal Screening Tool (MUST) ≥ 2 [41], any other severe clinical condition that would prevent optimal participation in the physical activities prescribed, as well as advanced age impeding the patient to adhere at the planned study follow-up period.

#### Methods of recruitment and random allocation

PCa patients will be randomized by a centralized computer process (Research Electronic Data Capture - REDCap® database platform) coordinated by IEO and assigned in a ratio 1:1 to one of the two arms: intervention group (IG) and control group (CG) using Randomization will be performed using. Study arms will be balanced taking into account the androgen deprivation therapy, pelvic lymph node involvements and surgery, in both centres. A progressive identification number will be assigned to each subject, and at randomization a link between the subject’s identification number and the arm will be established. Only those men who sign the informed consent form and the privacy disclosure, will be enrolled.

All data collected will be uploaded on dedicated electronic databases and will be treated with confidentiality, following the current privacy policy [42]. We will conduct the trial according to the ICH Good Clinical Practice (GCP) guidelines.

#### Study intervention

The principal goal of the intervention is to encourage the change of habitual diet and level of physical activity that may help in reducing or attenuating bowel and/or urinary problems during RT and to cope with feelings of anxiety or depression that this illness tend to engender. Interventions will be delivered by trained staff and participants will be followed up to 12 or 18 months depending on the arm (IG or CG, respectively). The baseline visit will be organized concurrently with the simulation TAC used to set up RT. Data will be collected in person and prospectively at each visit as reported in Table 1 and summarized in Supplementary Table 1.

**Table 1:** Study assessments

Assessment	Instruments	Visits				
		Baseline	After RT	T6	T12	T18^**b**^
Height^a^, weight, waist and hip circumference, Body Mass Index	Calibrated scales, stadiometer, tape measures	✓	✓	✓	✓	✓
Heart rate and blood oxygen saturation	Finger pulse oximeter	✓	✓	✓	✓	✓
Total, HDL, LDL cholesterol, triglycerides, glucose, insulin, PSA, and other serum biomarkers^c^	Blood Sample	✓		✓	✓	✓
Intestinal microbiome composition	Fecal Sample	✓	✓	✓	✓	✓
Body composition	BIVA (Bioelectrical Impedance Vector Analysis – Nutrilab device AKERN Srl – Italy)	✓	✓	✓	✓	✓
Food consumption	16-items Dietary Questionnaire	✓	✓	✓	✓	✓
Physical activity	International Physical Activity Questionnaire (IPAQ)	✓	✓	✓	✓	✓
Steps	Pedometer-like device (wrist band)	IG	–	CG	–	–
Quality of Life	Functional Assessment on Cancer Therapy (FACT-P)	✓	–	✓	✓	✓
Self-efficacy	Self-Efficacy Scale (GS-EF)	✓	–	✓	✓	✓
Anxiety	Anxiety Scale for Prostate Cancer (MAX – PC)	✓	–	✓	✓	✓
Life orientation	Life Orientation test (LOT-R)	✓	–	✓	✓	✓
Personality traits	Personal Traits Questionnaire (Con-OR)	✓	–	✓	✓	✓
Patient reported acute and late toxicity	Questionnaire acute and late toxicity	✓	✓	✓	✓	✓
Erectile function	International Index of Erectile Function (IEEF)	✓	✓	✓	✓	✓
Urinary function	International Prostate Symptoms Score (IPSS), International Consultation on Incontinence Questionnaire (ICIQ-SF)	✓	✓	✓	✓	✓
Acute and late toxicity	RTOG/EORTC^d^	✓	✓	✓	✓	✓

^a^- Height will be assessed only at baseline; ^b^ T18 visit is planned only for control group; ^c^ Other serum biomarkers: testosterone, estradiol, sex hormone binding globulin, high sensitive C-reactive protein (hs-CRP), adiponectin, 25-hydroxy vitamin D, Interleukin-6 (IL-6), Luteinizing hormone (LH); ^d^ Toxicity criteria of the Radiation Therapy Oncology Group (RTOG) and the European Organization for Research and Treatment of Cancer (EORTC)

#### Intervention group

Participants randomized to the IG will be offered individualized counseling based on their lifestyle habits to improve their dietary habits and physical activity levels. The intervention is provided by a dietitian and a physiotherapist.

At baseline, patients will be given individualized counseling based on patient’s dietary habits, to reduced amounts of insoluble fiber, to prefer foods rich in soluble fiber (for example wheat, corn, oats, rye, barley, legumes peeled, apple, carrots). In case of GI toxicities individualized indication will be given to reduce the assumption of lactose (milk and fresh cheese), caffeine and alcohol (low stimulant). Whether symptom remission has occurred, patient will be able to adhere to a more comprehensive and variable diet, based on World Cancer Research Fund (WCRF) recommendations [43]. Briefly, they recommend maintaining body weight in the normal range, engage daily physical activity and limit sedentary activities, eat vegetables every day, limit daily consumption of energy-dense foods, sugary drinks, red meat and alcohol.

In the same time, the physiotherapist will provide individualized indications to improve genitourinary health and to advise about common RT side effect (urinary incontinence, erectile dysfunction and pelvic pain) [44, 45]. The physiotherapist will also provide hints to prevent and eventually manage the lymphedema of genitalia/lower limb for patients who underwent to pelvic lymph-node dissection, following the international recommendations [46]. This specialist will also encourage to get a sufficient level of physical activity. This goal could be reached improving the general fitness status of the patient, providing a tailored program according to his preferences and habits [47]. The program will be composed by both aerobic and anaerobic exercises [20]. Reasonably, the initial goal will be to plan and implement daily purposeful mild to moderate exercise for a minimum of at least 10 min/day with a step-wise increase in time and intensity. One of the easiest activities to promote is to walk at least 10.000 steps every day, according to patient’s capability. Participants will be invited to wear the pedometer and instructed to count the total number of steps, to improve their self-monitoring and reduce sedentary time. These advices will be adapted and matched with international recommendation [43, 48] during the 6-months intervention to ensure also positive long-term effects [49, 50].

Four/five face-to-face visits (depending on the arm) and two telephone calls will be planned over the study period (intervention and follow-up) to monitor the adherence to the intervention, to support the participants, to provide personalized hint to deal with side effects, and to repeat and reinforce strategies guidance (Fig. 2). Individualized goals will be verified at each contact and workable solutions will be proposed in case of specific problems [51]. Each goal will be stated and included in a concrete and verifiable outcome (reduction of fiber and alcohol; increased use of public transportation or walking to go to work; reduction of car use; increased use of stairs instead of the elevator).

#### Control group

At baseline, participants included in the CG will receive general advices and materials available for patients undergoing RT (Fig. 2). According to the cross-over design, the CG will cross to the intervention approach after 6-months, to actively enhance compliance towards suggested lifestyle recommendations, as proposed for the IG.

#### Endpoints of the study

The primary objective is to evaluate the effect of 6-months intervention measuring the different adherence to a healthy lifestyle score between groups (IG and CG). The score will be calculated according to the WCRF recommendations [43, 52]. The final score will range from 0 (minimal adherence) to 7 (maximal adherence) [53].

As secondary outcomes, we will measure the change from baseline in fasting serum metabolic and inflammatory biomarkers. Likewise, the change in microbiota/microbiome, “alpha e beta-diversity” will be examined, as well as the change in acute and late toxicity, patient urinary function, QoL, anxiety, body composition, during the study intervention will be further evaluated (Supplementary Table 1). The change in patient self-efficacy, self-mastery and self-esteem will be also analyzed from the baseline. In a subgroup of participants, the association between VDR polymorphisms, change in diet and serum biomarkers and microbiota composition will be also evaluated. The association between change in microbiome and serum biomarkers with gastrointestinal symptomatology and acute and late toxicity, according to Toxicity criteria of the Radiation Therapy Oncology Group (RTOG) and the European Organization for Research and Treatment of Cancer (EORTC) will be investigated.

In Supplementary Table 2 are reported the statistical consideration for the Sample size calculation and the analytic plan.

#### Serious adverse events

Participants will be monitored over the course of the study during the follow-up visits and motivation calls. If they do experience an adverse event, this will be brought immediately to the attention of the clinical staff. Moreover, body composition will be monitored to identify any harmful weight loss and any changes in mass and hydration. The periodic recording of blood oxygen saturation and HR could offer a constant evaluation of patient’s state and preventing hypoxaemia’s cases. Clinicians will evaluate participants’ physical condition and they will make a decision whether patients can continue the intervention or advise them to leave the study. Participants will also be monitored for injuries or problems associated with increased physical activity.

#### Ethical considerations and study registration

Ethical approval has been obtained from the Ethics Committee of the European Institute of Oncology (Reference number: n. R1372/20 – IEO-1442) and of the National Cancer Institute, “Fondazione G. Pascale”, Naples (Prot. N. 2/21). The study will be conducted in agreement with the Helsinki Declaration and with current legislation in the matter of handling of personal data. The trial has been retrospectively registered on December 13, 2021 at the ClinicalTrials.gov (NCT05155618).

## Discussion

We present a protocol of an intervention trial focused on dietary and physical activity counselling in a group of men undergoing RT for PCa in two Italian centers (Milan and Naples). This randomized two-arm crossover trial is innovative in its design as we propose a combined intervention program including both dietary and physical activity counselling for PCa patients undergoing RT, to improve QoL, by controlling side effects and to coping with feelings of anxiety and depression. Despite the lack of clear evidence, a specific dietary strategy, the intervention aims to improve intestinal health at an early stage, to trigger efficacy and long lasting gastrointestinal benefit. Patients with PCa have high incidence of depression and anxiety across the pre- and post-treatment period [54]. Men are less likely to discuss their physical or psychological concerns with health professionals and they avoid seeking psychological support [55]. The scheduled visits and the motivational interviewing approach chosen should ensure a more active role of the patients in lifestyle changes to achieve success. Motivational interviewing approach aims to enhance self-efficacy and personal control for behaviour change, using an interactive, empathic listening style to increase confidence and motivation in an open-ended discussion. This approach has proved to be effective for cancer patients who are experiencing treatment cancer related fatigue and it helps addressing health behaviours and psychosocial needs [56, 57].

Previous systematic reviews and meta-analysis demonstrated that exercise intervention for PCa patients improves cardiovascular fitness, fatigue, QoL and social and cognitive functioning [21, 29, 58]. More recently, a meta-analysis investigated the effect of exercise training on inflammatory profile and immune function [28]. Combining aerobic and resistance training, PCa survivors are likely to experience a small decrease in pro-inflammatory markers like TNF and CRP. The authors found a trend to decreased anti-inflammatory citokines, with a change in their ratios that may produce a more optimal anti-tumor environmental. We did not plan any structured physical activity, but our protocol will equip the patients with a pedometer device to quantify physical activity by means of a common and easily understood metric (i.e., steps). Pedometer-based walking interventions have demonstrated their effectiveness in increasing physical activity in adult populations [59]. Objective measuring of physical activity in addition to a standard measurement (questionnaire) can add further precision to the physical activity level reached by participants during the intervention.

In our trial, the investigation of changes in microbiota features and the interaction with cytokines and adipokines will help understanding the role of immune system. It has been demonstrated that the gut microbiota may contribute to the pathogenesis of radiation enteropathy and how it presents opportunity to predict, prevent or treat radiation enteropathy [60], but clinical studies on PCa patients and evidences regarding the interactions between diet, lifestyle and microbiota are lacking. MicroStyle trial aims to carry out a comprehensive molecular analysis to investigate the influence of irradiation on gut microbiota in PCa patients. Moreover we will also be able to evaluate whether the intervention will provide microbiota diversity and reduce side effects of RT. In addition, the 6-months follow-up allows the evaluation of the effect of the intervention when patients should have recreated a healthier microbiome and have less treatment side effects.

Due to the high incidence of PCa worldwide, and the potential gastrointestinal and genitourinary side effects of pelvic RT, there is the need for evidence regarding the most effective approach in promoting healthy dietary habits and lifestyle in patients undergoing RT for PCa. The crossover design will provide us the possibility to evaluate the best timing (during vs after the end of RT) of the intervention in term of controlling side effects and to promote healthy lifestyle according to international guideline [43, 48].

Few clinical trials have investigated the effect of diet and physical activity counselling on PCa patients undergoing different types of treatments (RT, androgen deprivation therapy, surgery). A recent review evaluating the effectiveness of nutritional interventions involving dietary counselling on GI toxicities in patients receiving pelvic RT [17] demonstrated a lack of published RCTs. According to the authors, it is still unclear which is the best nutritional approach for the management of GI toxicity, because the proposed nutritional approach differed among studies and results varied. Thus, RCT are warranted. An emerging evidence is that dietary fiber should provide a protective role to intestinal health after pelvic RT, mainly through its impact on the microbiota [61]. The intestinal microbiota sampled before pelvic RT seems to predict the outcome with regards to treatment-induced symptoms [36, 38]. Moreover, radiation induces dysbiosis and reduced microbial diversity, with toxicity correlating to diversity and certain bacterial profiles [13, 62].

## Conclusion

The role of the gut microbiota in the gastrointestinal toxicity of RT has obtained great interest and evidences regarding the most effective approach in promoting a reduction of toxicity through the adoption of a healthy lifestyle in PCa patients are warranted. The results of this innovative project will provide useful information for future interventions and holds promise to have a large public health impact for PCa survivors.

## Supplementary Information

**Additional file 1: Supplementary Table 1.** Measures collected over the course of the MicroStyle study.

**Additional file 2:** **Supplementary Table 2. **Statistical consideration for the Sample size calculation and the analytic plan.

## Data Availability

Data of this article will be not available until the final report of this study to avoid bias toward the analysis.

## Abbreviations

PCa: Prostate cancer
RT: radiotherapy
IG: Intervention Group
CG: Control Group
RC: randomized controlled trial
IMRT: Intensity-Modulated RT
QoL: quality of life
ECOG PS: Eastern Cooperative Oncology Group Performance Status Scale
BMI: body mass index
MUST: Malnutrition Universal Screening Tool
hs-CRP: C-reactive protein
IL-6: Interleukin-6
LH: Luteinizing hormone
RTOG: Radiation Therapy Oncology Group
EORTC: European Organization for Research and Treatment of Cancer
WCRF: World Cancer Research Fund
