# Supplementary material for: Life style and interaction with microbiota in prostate cancer patients undergoing radiotherapy: study protocol for a randomized controlled trial
Source: BMC Cancer. 2022 Jul 19;22:794. doi: 10.1186/s12885-022-09521-4 (PMC9295396; doi:10.1186/s12885-022-09521-4)
Supplement: Supplementary file 1 — Additional file 1: Supplementary Table 1. Measures collected over the course of the MicroStyle study. [file 12885_2022_9521_MOESM1_ESM.docx]

**LIFE STYLE AND INTERACTION WITH MICROBIOTA IN PROSTATE CANCER PATIENTS UNDERGOING RADIOTHERAPY: STUDY PROTOCOL FOR A RANDOMIZED CONTROLLED TRIAL**

Patrizia Gnagnarella^1^^^§^, Giulia Marvaso^2,3§^, Barbara Alicja Jereczek-Fossa^2,3^, Ottavio de Cobelli^3-4^, Maria Claudia Simoncini^5^, Luiz Felipe Nevola Teixeira^5^, Annarita Sabbatini^6^, Gabriella Pravettoni^3,7^, Harriet Johansson^8^, Luigi Nezi^9^, Paolo Muto^10^, Valentina Borzillo^10^, Egidio Celentano^11^, Anna Crispo^11^, Monica Pinto^12^, Ernesta Cavalcanti^13^, Sara Gandini^9^ for the MicroStyle Collaborative Group.

**Supplementary table 1**. Measures collected over the course of the MicroStyle study

| **Blood sample** | A fasting blood sample will be collected at each visits (except after radiotherapy) to measure PSA, insulin, testosterone, sex hormone binding globulin (SHBG), LH, and lipid profile (total, HDL, LDL cholesterol and triglycerides), glucose, hs-CRP, adiponectin, 25OHD. PSA, glucose and lipid profile will be analyzed on fresh blood sample as routinely done for these patients. Serum levels of insulin, and luteinizing hormone (LH), will be determined by a chemiluminescence microparticle immunoassay (CMIA). Serum concentrations of testosterone, 25-hydroxy-vitamin D, estradiol will be analyzed by chemiluminescence immunoassays designed for the IDS-iSYS Multi Discipline Automated System Analyser (Immunodiagnostic Systems Limited, UK). Adiponectin will be measured by an Enzyme linked immunoassay designed for the automated platform ELLA (ProteinSimple, Biotechne). |
| --- | --- |
| **Fecal sample** | Four/five fecal samples per patient will be self-collected by patients at each visits in a collection tube prefilled with preservative liquid to maintain the microbial DNA stability at room temperature, following the instructions delivered by staff member. Fecal samples will be further transported to IEO’s laboratory, who is the responsible for all the downstream process of gut microbiota analysis. After amplification of V3-V4 16S rRNA DNA regions, samples will be sequenced using Illumina platform sequencer at IEO’s laboratory. The related bioinformatic and statistical analysis will provide us the identification of the structure and composition of the microbial community, with the aim to identify a core microbiota related to the selected host treatment and/or the specific disease (1). Moreover, the identification of the complete feces’ metagenome will be performed by means of shotgun sequencing, improving taxonomic resolution and revealing at species level which are the specific taxa associated with the treatment and enteropathy. Finally, a correlation between variation in the luminal microbiota population and host metabolic pathway changes will be investigated. |
| **Anthropometric measurement** | Anthropometric measurement will be collected at each visits. Weight and height will be recorded without shoes to the nearest 0.1 kg and 0.1 cm, using a calibrated dedicated scale and a stadiometer, respectively, and then will be used to calculate BMI (kg/m^2^). Waist and hip circumference will be collected using a flexible tape and following the standard collection methods: waist circumference will be measured in correspondence of the iliac crest; hip circumference will be measured at the maximal circumference of the buttocks (2)**.** |
| **Body composition** | Body composition will be measured by bioelectrical impedance (BIVA) at all visits. BIVA (Nutrilab device, AKERN Srl – Italy) is a more accurate method for a quick measurement of body compartments(3–5). The direct analysis of the two components of the impedance vector (Z), resistance (R, Ohm) and reactance (Xc, Ohm), allows a semiquantitative evaluation of body composition in terms of body cell mass and hydration status. Data for total body water (TBW), body cell mass (BCM), extracellular water (ECW), fat-free mass (FFM), fat mass (FM) and percentage fat mass (% FM) will be available for all participants and will be used for identifying changes of fat and fat-free mass over the study period. |
| **Food consumption** | Food consumption will be measured at each visits using a short self-administered questionnaire(6) recently developed to assess adherence to the Mediterranean diet in the Italian population. In this contest, it will be used to record daily or weekly intake of the main food groups over the previous months and the change over the study. In this population, we decided to explore the consumption of dairy products, focusing also on cheese for the contrasting effect of different dairy products on cancer risk (i.e., protective for colon, risk for prostate) (7). |
| **International Physical Activity Questionnaire** | International Physical Activity Questionnaire (IPAQ) will be used at each visits to monitor levels of physical activity. This questionnaire is a validated method (8) that consists of questions that record the frequency and duration of mild, moderate, and strenuous exercise performed during free time in the previous 7 days measuring physical activity and inactivity. The total hours per week spent in each activity will be multiplied by the estimated metabolic cost of each activity (metabolic equivalent (MET) value) as determined from the Compendium of Physical Activities(9). |
| **Quality of Life** | Quality of life will be measured by FACT-P (Functional Assessment of Cancer Therapy – Prostate) questionnaire that contains scales and items addressing the functional aspects of QoL and symptoms that commonly occur in PCa patients. The 39-items questionnaire measures physical, social/family, emotional, functional well-being, relationship with doctor, prostate cancer symptoms on a 5-point scale (10). |
| **Life orientation test** | The revised life orientation test (LOT-R) will be used to measure the mental disposition/attitude to optimism/pessimism. It is a 10-item questionnaire and respondents rate each item on a 4-point scale (11). |
| **Self-efficacy** | An Italian adaptation of the General Self-Efficacy (GS-F) scale will be used to assess a general sense of perceived self-efficacy with the aim to predict coping with daily hassles as well as adaptation after experiencing all kinds of stressful life events (12). |
| **Anxiety** | The level of anxiety will be measured at all visits using the Italian version of the Memorial Anxiety Scale for Prostate Cancer (MAX-PC). This scale measures general anxiety and related to PSA levels, and fear of recurrence (13). |
| **Personality traits** | Personality traits will be measured using a validated questionnaire (14) (ConOR) that consists of 34 items scored on a 5-point likert scale and provides information on 5 dimensions: control on self, self-oriented, need to be confirmed/seen, oriented on others, control on relationship and others' emotional protection. |
| **Toxicity** | Evaluation of acute and late gastrointestinal toxicities will be scored according to Radiation Therapy Oncology Group and European Organization for Research and Treatment of Cancer (RTOG/EORTC) scoring criteria (15) and registered as the maximum toxicity observed during the follow-up. |
| **International Prostate Symptom Score** | The International Prostate Symptom Score (IPSS) will be used to measure the severity of lower urinary tract symptoms. The IPSS is a validate and reproducible scoring system made up of 7 questions related to voiding symptoms (16). |
| **International Index of Erectile Function** | The sexual function will be evaluated using the International Index of Erectile Function (IIEF), a widely used, multi-dimensional self-report instrument for the evaluation of male sexual function(17). |
| **International Consultation on Incontinence Questionnaire-Short Form** | The International Consultation on Incontinence Questionnaire-Short Form (ICIQ-SF) is a 4-item tool for a subjective measure for evaluating the severity of urinary loss and condition-specific quality of life (18). |

**References:**

1. Frugé AD, Ptacek T, Tsuruta Y, Morrow CD, Azrad M, Desmond RA, et al. Dietary Changes Impact the Gut Microbe Composition in Overweight and Obese Men with Prostate Cancer Undergoing Radical Prostatectomy. J Acad Nutr Diet. 2018;

2. Center for Health Statistics N. Anthropometry Procedures Manual. 2017;

3. Piccoli A, Rossi B, Pillon L, Bucciante G. A new method for monitoring body fluid variation by bioimpedance analysis: The RXc graph. Kidney Int. 1994;

4. Buffa R, Mereu E, Comandini O, Ibanez ME, Marini E. Bioelectrical impedance vector analysis (BIVA) for the assessment of two-compartment body composition. European Journal of Clinical Nutrition. 2014.

5. Norman K, Stobäus N, Pirlich M, Bosy-Westphal A. Bioelectrical phase angle and impedance vector analysis - Clinical relevance and applicability of impedance parameters. Clinical Nutrition. 2012.

6. Gnagnarella P, Dragà D, Misotti AM, Sieri S, Spaggiari L, Cassano E, et al. Validation of a short questionnaire to record adherence to the Mediterranean diet: An Italian experience. Nutr Metab Cardiovasc Dis. 2018;

7. WCRF/ AICR. Diet, nutrition, physical activity and cancer: a global perspective: a summary of the Third Expert Report. World Cancer Research Fund International. 2018.

8. Craig CL, Marshall AL, Sjöström M, Bauman AE, Booth ML, Ainsworth BE, et al. International physical activity questionnaire: 12-Country reliability and validity. Med Sci Sports Exerc. 2003;35(8):1381–95.

9. Ainsworth BE, Haskell WL, Herrmann SD, Meckes N, Bassett DR, Tudor-Locke C, et al. 2011 compendium of physical activities: A second update of codes and MET values. Medicine and Science in Sports and Exercise. 2011.

10. Esper P, Mo F, Chodak G, Sinner M, Cella D, Pienta KJ. Measuring quality of life in men with prostate cancer using the functional assessment of cancer therapy-prostate instrument. Urology. 1997;

11. Scheier M, Carver C, Bridges M. Self Report Measures for Love and Compassion Research: Optimism LIFE ORIENTATION TEST-Revised (LOT-R). J Personal Soc Psychol. 1994;

12. Sibilia Lucio SR& JM. Italian Adaptation of the General Self-Efficacy Scale [Internet]. website. 1995 [cited 2021 Nov 30]. Available from: http://userpage.fu-berlin.de/~health/italian.htm

13. Roth AJ, Rosenfeld B, Kornblith AB, Gibson C, Scher HI, Curley-Smart T, et al. The memorial anxiety scale for prostate cancer: Validation of a new scale to measure anxiety in men with prostate cancer. Cancer. 2003;

14. Mazzocco K, Masiero M, Monzani D, Milani A, Didier F, Pravettoni G. A disease prone personality: development and validation of the ConOr questionnaire. J Res Pers.

15. Cox JD, Stetz JA, Pajak TF. Toxicity criteria of the Radiation Therapy Oncology Group (RTOG) and the European organization for research and treatment of cancer (EORTC). International Journal of Radiation Oncology, Biology, Physics. 1995.

16. Barry MJ, Fowler FJ, O’Leary MP, Bruskewitz RC, Holtgrewe HL, Mebust WK, et al. The American Urological Association symptom index for benign prostatic hyperplasia. The Measurement Committee of the American Urological Association. J Urol. 1992;

17. Rosen RC, Riley A, Wagner G, Osterloh IH, Kirkpatrick J, Mishra A. The international index of erectile function (IIEF): A multidimensional scale for assessment of erectile dysfunction. Urology. 1997;

18. Avery K, Donovan J, Peters TJ, Shaw C, Gotoh M, Abrams P. ICIQ: A brief and robust measure for evaluating the symptoms and impact of urinary incontinence. Neurourology and Urodynamics. 2004.
