# Supplementary material for: Life style and interaction with microbiota in prostate cancer patients undergoing radiotherapy: study protocol for a randomized controlled trial
Source: BMC Cancer. 2022 Jul 19;22:794. doi: 10.1186/s12885-022-09521-4 (PMC9295396; doi:10.1186/s12885-022-09521-4)
Supplement: Supplementary file 2 — Additional file 2: Supplementary Table 2. Statistical consideration for the Sample size calculation and the analytic plan. [file 12885_2022_9521_MOESM2_ESM.docx]

**LIFE STYLE AND INTERACTION WITH MICROBIOTA IN PROSTATE CANCER PATIENTS UNDERGOING RADIOTHERAPY: STUDY PROTOCOL FOR A RANDOMIZED CONTROLLED TRIAL**

Patrizia Gnagnarella^1^^^§^, Giulia Marvaso^2,3§^, Barbara Alicja Jereczek-Fossa^2,3^, Ottavio de Cobelli^3-4^, Maria Claudia Simoncini^5^, Luiz Felipe Nevola Teixeira^5^, Annarita Sabbatini^6^, Gabriella Pravettoni^3,7^, Harriet Johansson^8^, Luigi Nezi^9^, Paolo Muto^10^, Valentina Borzillo^10^, Egidio Celentano^11^, Anna Crispo^11^, Monica Pinto^12^, Ernesta Cavalcanti^13^, Sara Gandini^9^ for the MicroStyle Collaborative Group.

| **Calculation of the target sample size** |
| --- |
| We estimate that about 300 non-metastatic PCa patients are treated every year at the two centres. According to our experience, we expect that approximately one third of the screened patients meets the eligibility criteria and are inclined to participate in intervention studies. The perception is that the trial treatment may offer them the opportunity to control cancer, extending length and quality of life, while obtaining high-quality medical care and follow-up (**Figure 2**).  Considering as main endpoint the percentage of adherent patients (defined as Healthy lifestyle score >4), a sample size of 150 patients per arm will allow us to reach a power of 80% and a 20% difference in the percentage of patients adhering to recommendation, assuming an adherence in the intervention group of 40% under the null hypothesis and 60% in the alternative hypothesis. The hypotheses in frequency of adherence are based on results found in a previous study in a similar setting (1,2).The statistical test is a two-sided Z test with 'pooled variance'. The level of significance considered is 0.01 in order to take into account multiple testing for exploratory analyses.  The score of adherence will be computed using BMI, physical activity and food consumptions, according to the standardized system (3). Briefly, WCRF recommendations (4) will be used. They specify that individuals should maintain body weight in the normal range, engage daily physical activity, eat vegetables every day, limit daily consumption of energy-dense foods, sugary drinks, red meat, and alcohol. The score will be computed assigning participants a score based on quantitative cutoffs according to information collected during the baseline and the follow-up visits (BMI; level of physical activity; food consumptions). A score of 1, 0.5, and 0 for complete, partial, and non-adherence will be assigned respectively. The final score will range from 0 (minimal adherence) to 7 (maximal adherence). |

| ***Statistical methods*** |
| --- |
| In order to describe patients recruited in the study, descriptive statistics (median, range interquartile, minimum e maximum for continuous variables and frequencies for categorical variables) will be reported by initial arm for demographic characteristics, baseline characteristics of the tumor, biochemical values, anamnesis and physical examination.  In order to profile microbiome and genomics, we will identify microbial taxa, anywhere on the tree of life, that are over- or under abundant and associated with change in diet and lifestyle. We will use a variety of tools based on log-linear regression models with negative binomial or zero-inflated Gaussian error models when dealing with counts data, and zero-inflated Beta models with relative data. Regression estimates will be FDR-corrected to account for the multiple hypothesis testing problem. Multivariate models will be used to control for confounding effects or to test hypotheses of the microbiome as a mediator between exposures related to lifestyle or diet and health outcomes. Other multivariate approaches, including PCoA and PLS-DA analysis, will be carried out. Alpha and Beta-diversity indexes differences will be compared using Wilcoxon rank test. We will employ the Data Integration Analysis for Biomarker Discovery (DIABLO) using Latent Components implementation in the mixOmics R package. The mixOmics block.splsda function will be used to identify the optimal number of components and taxa. We will also investigate how changes in time in beta-diversity is associated with change in diet and lifestyle. Because it is very challenging to find a suitable probabilistic distribution for the microbial data due to its unique features, such as zero-inflation, over-dispersion, complex correlation structure, and compositional nature (5,6), we will also carry out analyses based on the log-contrast model. In particular, the sparse linear log-contrast model (5) will be implemented to identify the taxa that are significantly associated with intervention, dietary factors or biomarkers. Taxa will be studied in the models also as response variables, as genetic association studies demonstrated that such inverse regression (treating dependent variables as covariates) is advantageous if there are multiple dependent variables and the distribution is difficult to specify (7). We will utilize the state-of-the-art compositional mediation analysis for microbiome data (R Package SparseMCMM) (8,9). The method enables us to estimate the total mediation effects of microbiome composition, as well as to select important microbial taxa mediating the diet-metabolite association and estimate taxon-specific mediation effects. Supervised heatmaps will be used to represent markers and taxa able to discriminate patients with good adherence to diet/lifestyle indications. We will report P-values and we will highlight associations that meet a FDR adjusted P-value less than or equal to 0.05 by the Benjamini and Hochberg method. |

**References**

1. Er V, Lane JA, Martin RM, Emmett P, Gilbert R, Avery KNL, et al. Adherence to dietary and lifestyle recommendations and prostate cancer risk in the prostate testing for cancer and treatment (ProtecT) trial. Cancer Epidemiol Biomarkers Prev. 2014;

2. McCahon D, Daley AJ, Jones J, Haslop R, Shajpal A, Taylor A, et al. Enhancing adherence in trials promoting change in diet and physical activity in individuals with a diagnosis of colorectal adenoma; a systematic review of behavioural intervention approaches. BMC Cancer. 2015;

3. Shams-White MM, Brockton NT, Mitrou P, Romaguera D, Brown S, Bender A, et al. Operationalizing the 2018 world cancer research fund/american institute for cancer research (WCRF/AICR) cancer prevention recommendations: A standardized scoring system. Nutrients. 2019;

4. WCRF/ AICR. Diet, nutrition, physical activity and cancer: a global perspective: a summary of the Third Expert Report. World Cancer Research Fund International. 2018.

5. Lin W, Shi P, Feng R, Li H. Variable selection in regression with compositional covariates. Biometrika. 2014;

6. Tang ZZ, Chen G. Zero-inflated generalized Dirichlet multinomial regression model for microbiome compositional data analysis. Biostatistics. 2019;

7. Majumdar A, Haldar T, Witte JS. Determining Which Phenotypes Underlie a Pleiotropic Signal. Genet Epidemiol. 2016;

8. Li Y, Yan H, Zhang Y, Li Q, Yu L, Li Q, et al. Alterations of the Gut Microbiome Composition and Lipid Metabolic Profile in Radiation Enteritis. Front Cell Infect Microbiol. 2020;

9. Wang C, Hu J, Blaser MJ, Li H. Estimating and testing the microbial causal mediation effect with high-dimensional and compositional microbiome data. Bioinformatics. 2020;
